# The cardio-oncology continuum: Bridging the gap between cancer and cardiovascular care

**DOI:** 10.21542/gcsp.2024.9

**Published:** 2024-01-03

**Authors:** Tanveer Shaik, Jill Bhavsar, Shreya Garg, Vasu Gupta, Sai Gautham Kanagala, Rohit Jain

**Affiliations:** 1 Avalon University School of Medicine, Willemstad, Curacao; 2 Government Medical College Baroda, Gujarat, India; 3 Dayanand Medical College & Hospital, Punjab, India; 4 Osmania Medical College, Hyderabad, India; 5 Avalon University School of Medicine, Willemstad, Curacao

## Abstract

Cancer and cardiovascular disease are two of the leading causes of death worldwide. Although cancer has historically been viewed as a condition characterized by abnormal cell growth and proliferation, it is now recognized that cancer can lead to a variety of cardiovascular diseases. This is due to the direct impact of cancer on the heart and blood vessels, which can cause myocarditis, pericarditis, and vasculitis. Additionally, cancer patients frequently experience systemic effects such as oxidative stress, inflammation, and metabolic dysregulation, which can contribute to the development of cardiovascular risk factors such as hypertension, dyslipidemia, and insulin resistance. It is important to closely monitor patients with cancer, especially those undergoing chemotherapy or radiation therapy, for cardiovascular risk factors and promptly address them. This article aims to explore the clinical implications of the underlying mechanisms connecting cancer and cardiovascular diseases. Our analysis highlights the need for improved cooperation between oncologists and cardiologists, and specialized treatment for cancer survivors.

## Introduction

Cardiovascular disease (CVD) and cancer are the two most prevalent and potentially fatal medical conditions worldwide^1^. In the United States, cancer ranks as the second-leading cause of death^2^, accounting for 8.8 million annual fatalities per year, compared to 17.7 million caused by CVD^3^ Given the enormous burden these diseases impose on humanity, cancer and CVD research have become top priorities for medical professionals, researchers, and data analysts worldwide. Over the past 25 years, the incidence rates of all malignancies combined have increased by 13%, and this figure is expected to rise by an additional 2% in the next 20 years^4^.

The presence of multiple comorbidities, such as hypertension (HTN) and diabetes, in cancer patients can significantly affect their clinical outcomes and care^5^. Although improvements in cancer care have resulted in increased chances of survival, treatment-related adverse effects have raised morbidity and mortality rates^6^, especially since the 5-year survival rate has significantly improved over the last 30 years^7^.

Despite the apparent differences between cancer and CVDs, mounting evidence suggests that they are biologically linked due to the negative effects of anticancer treatment on cardiovascular health and shared risk factors prevalent in the aging population^8^. Therefore, it is critical to recognize and treat cardiovascular (CV) complications in cancer patients^9,10^.

Chemotherapy and radiation therapy increase the risk of stroke, coronary artery disease, and other CVDs such as heart failure^11^. Cancer therapy can cause cardiotoxicity, which is the most common adverse effect that directly affects heart structure and function. In addition, accelerated development of cardiovascular disease (CVD) can occur, particularly when conventional CV risk factors are present^12^. Cancer can indirectly increase a person’s susceptibility to CVDs by causing inflammation, raising the risk of blood clots, and reducing immunological function^13^. Despite the significant impact of CVDs and cancer on global health, little research has been conducted on the connection between these two diseases. Clinical trials of anticancer therapies linked to CV damage usually lack identification of relevant cardiac outcomes^14^. Heart failure and cancer co-occur frequently, and the relationship between these two diseases is becoming increasingly evident^15,16^. Therefore, cardiologists’ involvement in managing cancer treatment-related CV side effects and assisting in overall cancer care from diagnosis to survivorship and beyond has become increasingly recommended. This approach is called cardio-oncology^17,18^. The objective of this paper is to examine the latest studies on the relationship between cancer and CVD, scrutinize the various mechanisms connecting these two diseases, and explore potential strategies for reducing the risk of CV complications in individuals with cancer. The ultimate goal is to enhance the treatment and outcomes of patients with both cancer and CVD, offering them optimism for a brighter future despite these serious health conditions.

**Figure 1.**
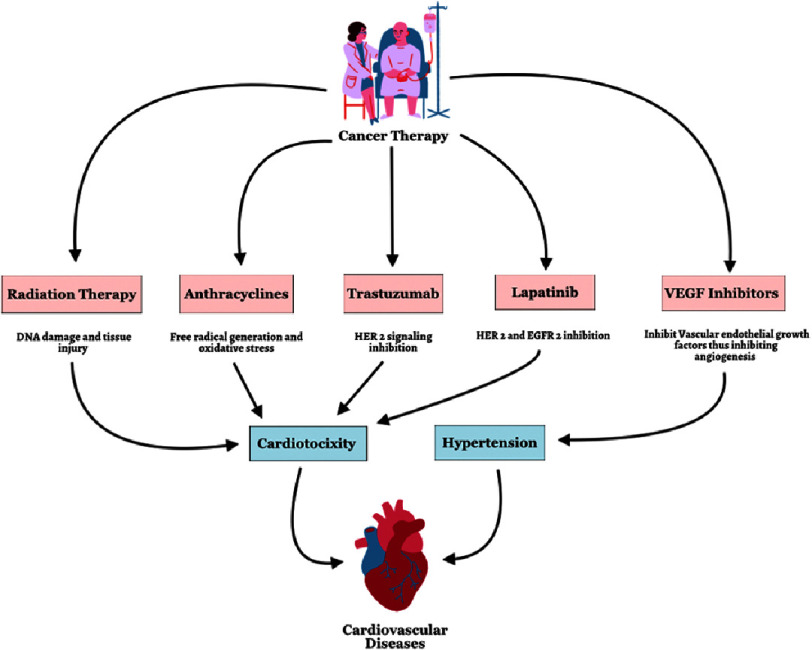
Cancer therapy leading to CVDs. HER 2 = human epidermal growth factor receptor 2; VEGF = vascular endothelial growth factor; EGFR = epidermal growth factor receptor.

### Adverse effects of cancer therapy

Cancer and its treatment modalities have long been recognized to induce CV functional decline in individuals with cancer, encompassing conditions such as left ventricular diastolic dysfunction, thromboembolic diseases, and HTN (Figure 1). Given the escalating prevalence of CV risk factors within our population, the additional harm caused by cancer therapies can have a detrimental impact on the CV health of those receiving therapy. Therefore, it is crucial to understand the diverse mechanisms underlying cardiac toxicities associated with prevailing cancer treatment modalities, as well as to explore various approaches to prevent or manage these risks.

Anthracyclines such as doxorubicin, daunorubicin, epirubicin, and idarubicin, are among the most widely used chemotherapeutic agents and have been shown to be effective against various cancers such as breast cancer, lymphoma, and other solid organ tumors^19^. They exert their chemotherapeutic effect by inhibiting the activity of topoisomerase-2, causing irreversible double-stranded breakages in genomic regions of active DNA synthesis, which eventually leads to cell apoptosis, preferentially in cancer cells that are actively proliferating as opposed to normal mitotic cells^20,21^. However, the emergence of cardiotoxicity, which adversely affects patient outcomes and severely restricts oncological treatment options, may compromise the clinical efficacy of chemotherapeutic agents. They have been proven to be highly cardiotoxic by generating free radicals and disrupting antioxidant defence mechanisms and cell repair^22^. Additionally, it disrupts the mitochondrial membrane structure, generates cardiotoxic cytokines, causes intracellular calcium overload, and uncouples the electron transport chain^22^. The detrimental effect of anthracyclines on cardiac cells is dose-dependent as they cause cell apoptosis at lower doses and necrosis as the dose increases, with more cases showing a decline in left ventricular ejection fraction within the first year after the end of treatment^23^. However, it depends significantly on the patient’s pretreatment left ventricular ejection fraction (LVEF) as early detection of decline in LVEF with periodic monitoring of cardiac function over time, and prompt initiation of heart failure treatment has been shown to improve the patient’s cardiac function^24^. The risk is higher in those who have undergone chest radiation, those who have received high anthracycline doses, and those with pre-existing CV risk factors such as HTN, obesity, diabetes mellitus, smoking, and dyslipidemia. Additionally, genetic changes in the ATP-binding cassette transporter (ABC) genes also influence anthracycline cardiotoxicity. The active cellular efflux of drugs, such as anthracyclines, is promoted by ABC genes, which contribute to multidrug resistance. If the activity of these genes is reduced, anthracycline accumulation within cells increases, leading to cardiotoxicity (Table 1)^25^.

**Table 1:** Adverse effects of chemotherapy and radiation therapy.

**Chemotherapeutic agents**	**Anthracyclines**	**HER2 Neu protein inhibitors**	**Lapatinib**	**Other HER2 receptor inhibitors**	**Radiation therapy**
**Mechanisms of Cardiotoxicity**	topoisomerase-2 inhibition, DNA damage, free radical generation, antioxidant disruption, mitochondrial dysfunction, and Ca overload	Decline in contractile activity of cardiomyocytes, Minimal signs of reduced LVEF and heart failure	Targets intracellular tyrosine kinase domain, impacting ligand-induced and ligand-independent HER 2 signaling pathways	Neratinib, Afatinib, Pertuzumab	Free radical damage, Microvascular and macrovascular injury, Valve stenosis
**Targeted Cancers**	Breast cancer, lymphoma, solid organ tumors	HER 2 Neu receptor-positive breast cancers	Advanced HER 2 Neu-positive breast cancer post prior anthracycline and trastuzumab treatment.	HER 2 Neu-positive breast cancer	Breast cancer (adjunctive treatment)
**Cardiotoxic Effects**	Cell apoptosis at lower doses, necrosis at higher doses	Minimal signs of reduced LVEF and heart failure	Less likely to cause cardiotoxic adverse effects than trastuzumab	Lower cardiotoxicity observed	Increased risk of CVD
**Reversibility**	Irreversible	Reversible	Reversible	Reversible	N/A
**Dose-Dependence**	Yes	No	No	No	N/A
**Impact on Cardiac Function**	Decline in left ventricular ejection fraction (LVEF) within the first year after treatment	Minimal signs of reduced LVEF and heart failure	Minimal signs of reduced LVEF and heart failure	Minimal signs of reduced LVEF and heart failure	pericarditis, valvular defects, coronary stenosis, and left anterior descending coronary artery stenosis
**Monitoring Recommendations**	Periodic monitoring of cardiac function, prompt initiation of heart failure treatment	Discontinue if LVEF decline >15% or LVEF falls to 50% or lower at any time during treatment	Periodic monitoring of cardiac function	Periodic monitoring of cardiac function	Regular CV monitoring and management of cardiac risk factors
**Risk Factors**	Female patients with prior chest radiation, high anthracycline doses, and CV risk factors (hypertension, obesity, diabetes, smoking, dyslipidemia)	Combination with anthracyclines increases incidence of heart failure and symptomatic LVEF decline	N/A	N/A	N/A
**Genetic Influence**	Genetic changes in ABC genes increase anthracycline accumulation leading to cardiotoxicity	N/A	N/A	N/A	N/A

**Notes.**

HER 2 human epidermal growth factor receptor 2 CVD cardiovascular diseases N/A not applicable LVEF left ventricular ejection fraction

Another class of chemotherapeutic drugs that have proven to be cardiotoxic are HER2/neu protein inhibitors, which include taxanes and monoclonal antibodies like trastuzumab. They are widely used in the treatment of HER2/neu receptor-positive breast cancers, which account for nearly 15–20% of invasive breast tumors. Overexpression of the HER2/neu receptor, responsible for regulating cell survival, leads to increased cell proliferation and growth^26^. Trastuzumab, a monoclonal antibody that targets the HER2/neu protein’s extracellular domain, is effective in treating HER2/neu receptor-positive breast cancer^27^. Trastuzumab therapy was previously considered generally safe and well-tolerated^28^, however advances in research and patient survival rates later illustrated the cardiotoxic effects of trastuzumab, which unlike anthracycline cardiotoxicity, is not dose-dependent and is reversible^23^. It causes a decline in contractile activity of cardiomyocytes rather than necrosis and alone shows minimal signs of reduced LVEF and heart failure.

To ensure the safety of patients receiving trastuzumab, discontinuation of the drug is recommended if there is a decline in LVEF of >15% or if the LVEF falls to 50% or lower at any time during treatment. It is important to note that patients who receive trastuzumab alone have a lower incidence of heart failure and symptomatic LVEF decline compared to those who receive it in combination with anthracyclines, and hence require less frequent cardiac monitoring (Table 1)^27,29,30^.

Lapatinib HER2 receptor inhibitor that inhibits the HER2 receptor’s intracellular tyrosine kinase domain and can act on both ligand-induced and ligand-independent pathways of HER2 signaling, can be given in patients with advanced and aggressive HER2/neu-positive breast cancer who have undergone previous anthracycline and trastuzumab treatment.

According to recent research, lapatinib is less likely to cause cardiotoxic adverse effects than trastuzumab^31^. Other HER2 receptor inhibitors, such as neratinib, afatinib, and pertuzumab, have also demonstrated lower cardiotoxicity when administered alone or in combination with trastuzumab^32^. Radiation therapy has significantly reduced the risk of cancer recurrence, however, research indicates that radiation therapy increases the risk of developing various CVD^33^. The underlying pathologies in the pericardium and myocardium that contribute to these CV events include free radical damage, microvascular and macrovascular injury, and valve stenosis, which typically manifests 10-15 years post-radiation exposure^34^.

Studies have also shown that women who received radiation therapy for breast cancer have a significantly higher risk of developing pericarditis, valvular heart defects, and coronary stenosis^35^. Women undergoing radiation therapy for left breast cancer exhibit a higher occurrence of stenosis in the mid and distal portions of the left anterior descending coronary artery than those undergoing radiation for right breast cancer^33^ and this correlation cannot be accounted for by factors such as pre-existing heart disease, underlying cardiac risk factors, or radiation exposure^36^ (Table 1).

HTN is also documented as the most common severe adverse event in patients with cancer receiving chemotherapy^37^ and is also one of the major risk factors contributing to CVDs such as ischemic heart disease, stroke, and heart failure, as well as kidney disease^38^. Cancer therapies cause HTN through a variety of mechanisms including direct effects on vascular endothelial cells or indirect renal effects and are most notably seen with vascular endothelial growth factor (VEGF) inhibitors such as, tyrosine kinase inhibitors, proteasome inhibitors, calcineurin inhibitors and adjunctive therapies like corticosteroids, exogenous erythropoietin, and non-steroidal anti-inflammatory drugs^39^.

VEGF signaling pathway inhibitors (VSPI) such as bevacizumab, sorafenib, and sunitinib are widely used in terminating tumors such as renal, hepatocellular, thyroid, and gastrointestinal stromal tumors, and are associated with cardiovascular comorbidities such as HTN, with a reported incidence of 20–90%^40^. They act by inhibiting the VEGF receptors on the endothelial cells, thus inhibiting angiogenesis and depriving the tumorous growth of oxygen and nutrient supply^41^. VEGF inhibitors have been documented to decrease the left ventricular diastolic function, thereby directing an increase in systemic vascular resistance (SVR) to be the cause of elevated blood pressure which is a product of cardiac output and SVR^42^. Antiangiogenic VSPIs can cause an increase in SVR due to a decrease in the density of microvessels, downregulation of nitric oxide synthesis^43^, and increasing concentrations of vasoconstrictors, such as endothelin-1, which contributes to an increase in vascular resistance and hence causes HTN. Through their indirect effect on renal endothelial cells and podocyte VEGF expression, VSPIs also lead to thrombotic microangiopathy and thereby HTN^44^. They are associated with increased synthesis of reactive oxygen species including H_2_O_2_, and O^2−^, causing vascular oxidative stress^45^ and contributing to the cardiotoxic profile of VSPIs (Table 2).

**Table 2:** Chemotherapy-related hypertension.

**Chemotherapy-Related Hypertension**	**Mechanisms**
**Chemotherapy Agents**	Vascular endothelial growth factor inhibitors (VEGF): Bevacizumab, Sorafenib, Sunitinib; tyrosine kinase inhibitors, proteasome inhibitors, calcineurin inhibitors, corticosteroids, exogenous erythropoietin, non-steroidal anti-inflammatory drugs
**Cardiovascular Comorbidities**	Ischemic heart disease, stroke, heart failure, kidney disease
**Mechanisms of Hypertension**	Direct effects on vascular endothelial cells, indirect renal effects, decreased left ventricular diastolic function, increased systemic vascular resistance (SVR), thrombotic microangiopathy, and increased synthesis of reactive oxygen species
**Reported Incidence of HTN**	20–90% with VEGF inhibitors
**Clinical Implications**	Increased risk of cardiovascular comorbidities and complications associated with hypertension
**Targeted Tumors**	Renal, hepatocellular, thyroid, gastrointestinal stromal, and others

**Notes.**

VEGF vascular endothelial growth factor SVR systemic vascular resistance

A meta-analysis of five randomized controlled studies found that the use of fibrosarcoma B-type (BRAF)/mitogen-activated kinase (MEK) inhibitors, such as vemurafenib-dabrafenib and encorafenib-trametinib, in the treatment of BRAF-mutant melanoma and BRAF-mutant colorectal cancer was also linked to hypertension, with a reported incidence of 19.5%^46^. The claimed mechanism of action is linked to a decrease in nitric oxide generation and an increase in CD47 in melanoma cells cultured *in vitro*, which blocks nitric oxide/cyclic guanosine monophosphate signaling (NO/cGMP) *via* thrombospondin-1^46^.

## Discussion

The probability of death due to CVD in individuals with cancer is influenced by multiple factors, including age at diagnosis, sex, race, primary cancer stage, year of diagnosis, and use of surgery^47^. With the declining cancer-specific mortality rate and aging of the surviving population, there is an increasing overlap between patients with heart disease and cancer^48^. Recent statistics show that the number of cancer survivors in the United States has exceeded 16.7 million^49,50^. The American Heart Association (AHA) has systematically evaluated and classified scientific data into clinical practice guidelines to improve CV health^51^.

In recognition of the emerging field of cardio-oncology, the National Comprehensive Cancer Network (NCCN) also offers guidelines on the stages, treatment, and surveillance of cardiomyopathy in cancer patients^52^. A study analyzed 7,529,481 cancer patients to investigate deadly cardiac disease in cancer and found that 5.24% (394,849) of them died from the disease (Table 3)^47^. The mortality rate due to heart disease in all cancer patients was 10.61 per 10,000 person-years, and the SMR for fatal heart disease was 2.24 (95% CI: 2.23, 2.25, relative risk: *p* = 0.0001), as shown in Table 3^47^.

**Table 3:** Cardiovascular mortality rates in cancer patients.

**Study**	**Number of cancer patients analyzed**	**Number of deaths from cardiac disease**	**Specific mortality rate for heart disease (per 10,000 person-years)**	**SMR for fatal heart disease (95% CI)**
**Stoltzfus 2020**	7,529,481	394,849	10.61	2.24 (2.23,2.25)
**Yao H 2023**	12,058	477	N/A	3.23 (2.97,3.52)

Among the various types of cancer, lung, breast, prostate, and colorectal cancer patients are at a higher risk of dying from fatal heart disease than patients with other types of cancer^47^. In the case of patients with gastroenteropancreatic neuroendocrine neoplasms, the risk of CVD was 1.2 times greater than that of the general population in the United States^53^. A study found that among the GIST patients identified between 2000 and 2019, 477 (4.0%) died from CVD, and their risk of CV mortality was 3.23 times higher than that of the US population, with a 95% CI of 2.97−3.52 (Table 3)^54^. Medical conditions with the highest SMR were aortic aneurysm and dissection, with an SMR of 3.58 and a 95% CI of 1.54−7.04, followed by cerebrovascular disorders, with an SMR of 3.28, a 95% CI of 2.62−4.04, and various illnesses of the arteries, arterioles, and capillaries, with an SMR of 10.35, and a 95% CI of 2.82–26.49 (Table 4)^54^.

**Table 4:** Standard mortality rate (SMR) for various cardiovascular diseases found in cancer patients.

**Study**	**Medical condition**	**SMR**	**95% CI**
**Yao H 2023**	Aortic aneurysm and dissection	3.58	1.54–7.04
**Yao H 2023**	Cerebrovascular disorders	3.28	2.62–4.04
**Yao H 2023**	Various illnesses of the arteries, arterioles, and capillaries	10.35	2.82–26.49

**Figure 2.**
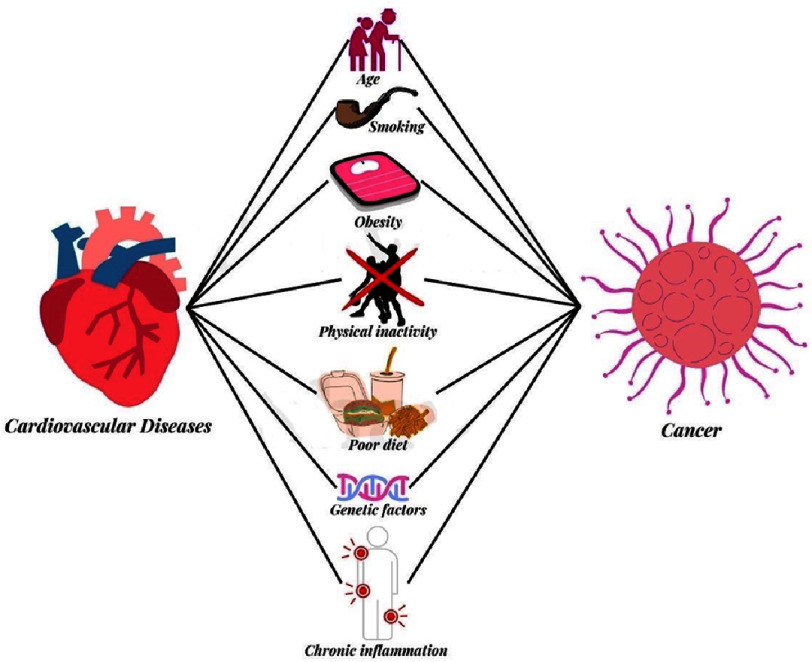
Common risk factors associated with CVD and cancer.

Smoking, HTN, obesity, and genetic predispositions are risk factors associated with an increased likelihood of both cancer and CVD (Figure 2)^55^. Aberrant Wnt signaling is a crucial factor in the pathophysiology of atherosclerosis and several malignancies^56,57^. Various genetic risk factors for cancer and CVD have been identified, and reports suggest that mutations in LRP6, a Wnt binding protein, may cause CVD and other cancers^58^. In addition, acquired mutations in DYRK1B, which is overexpressed in many types of tumors and has a functional mutation associated with CV disease, support the genetic connection between heart failure and cancer^59,60^. Nicotine, one of the harmful pathways activated by smoking, has been implicated in the pathogenesis of both cancer and CVD^61,62^. Patients with pre-existing CVD are at risk of developing smoking-related cancers^63^.

Obesity, a risk factor for one in five cancer types, has also been linked to CVD and cancer^64^. Adipose tissue is a significant source of estrogen, making women more susceptible to hormone-driven malignancies such as breast and ovarian cancers^65^. Obese individuals are at an increased risk for developing heart failure but may have a survival advantage, a phenomenon currently under investigation in cancer patients^66,67^. HTN was found to have the highest incidence among stomach and ovarian cancer patients, with over 30% of the 25,000 cancer patients diagnosed with HTN in a retrospective cohort (Table 5)^68^. In comparison to periods when chemotherapy was not administered, treatment with cytotoxic, targeted, or combination chemotherapy was associated with an average hazard ratio between a 2- and 3.5-fold increase in the risk of any degree of HTN^59^.

**Table 5:** Incidence of Hypertension in patients with stomach and ovarian cancer.

**Study**	**Cancer type**	**Number of cancer patients analyzed**	**Percentage with hypertension**
**Fraeman KH 2013**	Stomach	25,000	Over 30%
**Fraeman KH 2013**	Ovarian	25,000	Over 30%

Chemo- and radiation-induced cardiotoxicity are becoming more prevalent in the present scenario as these modes of treatment are the primary options for most cancer treatment regimens (Table 6). Chemotherapy can cause two types of cardiac toxicities: type 1 chemotherapy-induced cardiotoxicity and type 2 chemotherapy-induced cardiotoxicity, as stated in a study^69^. Type 1 chemotherapy-induced cardiotoxicity is characterized by cardiomyocyte damage, with anthracyclines being the prototypical drug class. In contrast, type 2 chemotherapy-induced cardiotoxicity, which is characterized by treatment-induced cardiotoxicity and the absence of structural abnormalities that are reversible upon therapy termination, is exemplified by trastuzumab (herceptin), according to the same study^69^. However, recent research using cardiac magnetic resonance imaging (MRI) has shown that the outlined classification pattern may not be as rigid as previously believed, as scar formation has been detected in patients presumed to have type 2 cardiotoxicity, and appropriate heart failure therapy improved the presumed type 1 cardiotoxicity^70,71^.

**Table 6:** Various cardiovascular diseases linked with cancer therapies.

**Cancer treatment**	**Cardiovascular diseases**	**Medications**
**Chemotherapy**	Hypertension, heart failure, arrhythmias	Beta-blockers, ACE inhibitors
**Radiation therapy**	Inflammation, scarring	Statins
**Anthracyclines**	AIC	Dexrazoxane
**Targeted therapies (e.g., trastuzumab)**	Trastuzumab-induced cardiotoxicity	ACE inhibitors and beta-blockers

VEGF plays a significant role in angiogenesis, endothelial cell survival, vasodilation, and cardiac contraction^72^. HTN is the most common CV adverse effect of VEGF-targeted angiogenesis inhibitor therapy, indicating the importance of VEGF and related signaling pathways in blood pressure regulation^73^. The hypertensive effect of VEGF-targeted therapy seems to be dose-dependent, since it is more frequently observed in patients receiving a higher dose of anti-VEGF cancer therapy, as reported in previous studies^74,75^.

In the treatment of males with advanced testicular cancer, vascular damage is one of the most significant long-term side effects of cisplatin-based chemotherapy^76^. The main CVD that has been researched are Raynaud’s phenomenon, early atherosclerosis, dyslipidemia, HTN, coronary artery disease, and thromboembolic events^77^. Patients who survive testicular cancer often develop CVD risk factors, such as obesity, HTN, hyperlipidemia, and diabetes mellitus^78^.

Owing to the increasing incidence of thoracic tumors and prolonged patient survival, radiation-induced heart disease has become more prevalent. Radiation has an impact on every aspect of the heart, causing subtle histopathological alterations or overt clinical illness^79^. The most typical type of involvement involves the pericardium and comprises constrictive pericarditis and asymptomatic pericardial effusion^79^. Compared to women without breast cancer, women with breast cancer had a higher incidence of CVD events, a higher mortality rate linked to CVDs, and a higher overall mortality rate^80^. The rate of major coronary events increased by 7.4% per gray when the heart was accidentally exposed to breast cancer radiation therapy^81^. This increase is inversely correlated with the mean dosage to the heart, which begins a few years after exposure and lasts for at least 20 years^81^.

In view of the aforementioned factors, there is an increasing need to address and provide recommendations with suitable scoring systems to identify patients with cancer who are at a high risk of CVD-related morbidity and mortality. The American Heart Association has made a strong case for the value of cardiac rehabilitation programs for cancer patients to mitigate CV risk and has recommended the use of the CORE (cardio-oncology rehabilitation) algorithm to recognize high-risk patients^48^. According to AHA/ACC, referring patients with acute coronary syndromes to cardiac rehabilitation programs has always been a Class I recommendation because it improves patients’ functional capacity through supervised exercise programs, facilitates CV risk reduction by offering appropriate counseling, lowers the rate of recurrent hospitalizations and, as a result, lowers CV morbidity and mortality^48^. The same strategy, when implemented properly with a multimodal collaboration between oncologists, cardiologists, and primary care physicians, can help reduce the CVD risk in cancer patients as well.

A multimodal collaboration, with treating providers working together to evaluate patients’ CV risk in a timed fashion, taking appropriate actions, can undoubtedly be a solution to this problem. Patients’ baseline CV risk should be evaluated before the onset of cancer therapy using a detailed history, thorough physical examination, and supplementary diagnostic modalities like ECG or echocardiography^69^. Once treatment has started, it must be determined which patients need to have CV follow-up. Following cancer treatment, follow-up recommendations should be tailored to the patient’s individual CV risk and comorbidity profile, specific anticancer therapy used, the overall survival prognosis of the underlying malignancy, and adverse cardiac effects during treatment.

Long-term cardiac surveillance programs are highly recommended for patients with breast cancer, lymphoma, and individuals who have had mediastinal radiation therapy^69^. AIC is frequently irreversible if not detected early and can result in progressive end-stage heart failure despite the use of medical therapy based on heart failure guidelines (Table 6)^73^.

It is predicted that more cancer survivors will develop chemotherapy-induced cardiomyopathy and require orthotopic heart transplantation because more recent chemotherapy drugs have demonstrated potential cardiotoxicity and cancer mortality is declining^82^. Trastuzumab withdrawal or discontinuation (4–8 weeks) can cure systolic dysfunction in the early phases of trastuzumab-induced cardiotoxicity, allowing the initiation of normal heart failure treatment (ACE inhibitors and beta-blockers) (Table 6)^83^. Although there is disagreement regarding the precise recovery from trastuzumab-induced cardiotoxicity, most patients report improved health following trastuzumab withdrawal along with the management of cardiac symptoms^84^.

Despite emerging data showing that CV treatment can improve both cardiac-specific and cancer-specific outcomes, cardio-oncology patients continue to receive inadequate CV care, although cardio-oncology services are becoming more prevalent in academic centers and local communities^85,86^. Another requirement for cardio-oncology programs is prompt access to testing and consulting, as both delayed treatment of cardiac issues and non-adherence to recommended cancer treatments are linked to unfavorable results^87^. Additional research is urgently required to determine when invasive treatments such as revascularization are beneficial given the extreme prevalence and coexistence of the two disease processes^88^. The utilization of a cardio-oncology service line, which integrates crucial infrastructure components based on a standardized system of care, is a practical and efficient care model for enhancing cardio-oncology care quality, patient access, and health equity in sizable, multi-hospital health systems^89^.

## Conclusion

To effectively prevent and treat cancer and CVD, it is crucial to understand the relationship between these two diseases. Cancer patients are more likely to develop CVD, such as heart failure, coronary artery disease, and cardiomyopathy, due to CV issues. This risk is particularly high for patients undergoing chemotherapy, radiation therapy, and targeted therapies for cancer treatment. The association between CVD and cancer is complex and involves intricate underlying mechanisms. Although the exact mechanisms remain unclear, vascular damage, oxidative stress, and inflammation are believed to play significant roles. Moreover, certain cancer treatments can cause cardiotoxicity, leading to impaired cardiac function and increased risk of CV events. These outcomes have important implications for therapy, as patients with cancer are more likely to experience CV problems even years after their cancer treatment has ended. This underscores the importance of careful monitoring and follow-up care for cancer survivors, with a focus on early identification and treatment of CV issues.

More collaboration is needed between oncologists and cardiologists, and cardio-oncology should be developed to address the unique needs of cancer patients with CVD. This collaboration should include the development of risk assessment tools and treatment guidelines tailored to this population as well as the investigation of innovative interventions and treatments for preventing and managing CV complications in cancer patients. The significant impact of cancer on CV health highlighted in this review underscores the importance of increasing awareness, working collaboratively, and providing specialized care for those at risk. Through continued research and collaboration, we can improve the outcomes and quality of life of cancer survivors, ultimately reducing the burden of CVD associated with cancer.

## Declarations

## Funding sources

The authors did not receive support from any organization for the submitted work.

## Competing interests

The authors have no competing interests to declare.

## Ethics approval and consent to participation

Not applicable

## Consent for publication

Not applicable

## Availability of data and material

All data were collected from articles published in Google Scholar and PubMed.

## Acknowledgement

Declared none.

## Disclosures

None.

## Authors’ contribution statement

Rohit Jain and Shreya Garg conceived the review article. Tanveer Shaik, Jill Bhavsar, Shreya Garg, and Vasu Gupta performed literature research and data analysis and assisted in deciding the article concept and design. Tanveer Shaik, Jill Bhavsar, Shreya Garg and Gautham Kanagala created the first draft of the article and then all the authors assisted in revision of the manuscript for important intellectual content, and final approval of the version. All authors have critically reviewed and approved the final draft and are responsible for the content and similarity index of the manuscript.

## Abbreviations

AIC: Anthracycline-induced cardiotoxicity
ABC transporter: ATP-Binding cassette transporter
CVD: Cardiovascular disease
CV: Cardiovascular
EGFR: Epidermal growth factor receptor
GIST: Gastrointestinal Stromal Tumors
HER 2: Human Epidermal Growth Factor Receptor 2
LVEF: Left ventricular Ejection fraction
HTN: Hypertension
VEGF: Vascular endothelial growth factor
